# Archaeal Enzymes and Applications in Industrial Biocatalysts

**DOI:** 10.1155/2015/147671

**Published:** 2015-09-30

**Authors:** Jennifer A. Littlechild

**Affiliations:** Henry Wellcome Building for Biocatalysis, Biosciences, College of Life and Environmental Sciences, Stocker Road, Exeter EX4 4QD, UK

## Abstract

Archaeal enzymes are playing an important role in industrial biotechnology. Many representatives of organisms living in “extreme” conditions, the so-called Extremophiles, belong to the archaeal kingdom of life. This paper will review studies carried by the Exeter group and others regarding archaeal enzymes that have important applications in commercial biocatalysis. Some of these biocatalysts are already being used in large scale industrial processes for the production of optically pure drug intermediates and amino acids and their analogues. Other enzymes have been characterised at laboratory scale regarding their substrate specificity and properties for potential industrial application. The increasing availability of DNA sequences from new archaeal species and metagenomes will provide a continuing resource to identify new enzymes of commercial interest using both bioinformatics and screening approaches.

## 1. Introduction

The application of enzymes in “White Biotechnology” for the synthesis of industrially important chiral compounds is becoming increasingly important for the pharmaceutical industry. Many companies who were traditionally not incorporating biocatalysis in their drug production programmes are now very keen to develop the technology. Enzyme chemistry can make reactions feasible which are currently unavailable using conventional methods. Use of enzymes for chemical processes is a route to lower energy consumption and reduced waste generation. In addition, the selectivity of enzymatic processes reduces the raw material costs and the safety issues surrounding the production of wasteful biproducts. It is anticipated that optimised enzyme production through further bioprocess intensification will lead to more economically viable and cost effective, sustainable compound production.

The wealth of genome and metagenome data now available makes searching for enzymes using both advanced bioinformatic and substrate screening approaches an area for development. Also more representatives of the different classified enzyme groups are being investigated for their application in industrial biocatalytic processes. The enzyme process is often used in a “cascade reaction” with traditional chemistry synthetic steps. When “nonnatural” industrial substrates are presented to enzymes found in “nature” it has been found that different classes of enzyme can use the same nonnatural compound as a substrate to carry out a specific biotransformation. This makes it difficult to predict which class of enzyme should be best for the biocatalytic process. Also using the enzyme in the presence of solvents or at nonphysiological pH can result in “side reactions” which are different from the normal activity of the enzyme catalyst.

The development of novel, efficient, and cost effective biocatalytic processes in a variety of industries is currently limited by the number of robust, highly selective, and useful biocatalysts. This paper will concentrate on specific novel enzymes from the archaeal kingdom that have been isolated from thermophilic marine and terrestrial environments. Thermophilic enzymes from archaea offer additional novelty in relation to those from thermophilic bacteria since they have been shown to be more primitive enzymes. An example of this is the* Sulfolobus solfataricus* Glyceraldehyde phosphate dehydrogenase (GAPDH) [1] which has the catalytic cysteine on the same secondary structure as other bacterial and eukaryotic ADHs but other residues involved in catalysis are presented into the active site from different secondary structural elements. This enzyme has only 18% sequence identity to other well-characterised GAPDHs and was thought to have a different overall structure until the crystal structure of the archaeal enzyme was determined. Some archaeal enzymes have evolved by a different route to their bacterial or eukaryotic equivalents in so much that they are a combination of different enzymes such as the L-aminoacylase from* Thermococcus litoralis* [2] that has only L-aminoacylase activity but is related regarding its sequence similarity to a carboxypeptidase enzyme from a* Sulfolobus* species.

Many of the archaeal species have novel metabolic pathways that are not found in other kingdoms of life. For example, some utilise modified versions of the canonical Embden Meyerhof and Entner-Doudoroff pathway involving a large number of novel enzymes [3] and have unusual pentose degradation pathways.

Some archaeal enzymes are more promiscuous in their activity than comparable enzymes from bacteria or eukaryotes: for example, the* S. solfataricus, Picrophilus torridus *glucose/galactose dehydrogenase dehydratase, and gluconate/galactonate dehydratase [4–6]. The archaeon* S. solfataricus* has a promiscuity in 2-keto-3-deoxygluconate aldolase from the Entner-Doudoroff pathway which is able to cleave KDG and D-2-keto-3-deoxygalactonate (KDGal) to produce pyruvate and D-glyceraldehyde. The aldolase also exhibits a lack of stereoselectivity in the reversible condensation reaction of pyruvate and D-glyceraldehyde. An understanding of the structural basis of the promiscuity has been studied [7].

Enzymes isolated from thermophilic archaea usually are more stable to high temperature, presence of solvents, and resistance to proteolysis which together are ideal features for industrial applications. Stability of an enzyme is dependent on maintenance of a functional structure, and the stability of any protein is marginal and equivalent to a small number of molecular interactions [8]. This remains the case with a thermostable protein, the only difference being that the free energy of stabilisation is slightly higher than that of its mesophilic counterpart [9]. The active form of a protein is usually held together by a combination of noncovalent forces including hydrogen bonds, ion pairs, hydrophobic bonds, and Van der Waals interactions. When these interactions are disrupted, for example, by elevated temperatures, both mesophilic and thermophilic proteins unfold into inactive but kinetically stable structures. Once unfolded in this manner the protein is prone to aggregation and chemical modification. Aggregation occurs when the hydrophobic residues of a protein that have been exposed by the polypeptide chain unfolding interact with hydrophobic residues from other protein molecules, which usually follows immediately after unfolding. Chemical modifications of the protein can include cysteine oxidation, deamination of asparagine and glutamine residues, and peptide bond hydrolysis. The unfolding of the protein may be reversible for smaller proteins but is usually irreversible with larger proteins.

Although the optimal temperature for activity of the thermostable archaeal enzymes is above what would normally be used in an industrial process they can be used at lower temperatures and usually maintain at least 20% of their maximum activity at ambient temperature. There is often an advantage to running a process at temperatures between 50 and 60°C since at this temperature many nonnatural substrates which are insoluble at room temperature become soluble at the higher temperature. The temperature for operation of the industrial process needs to be balanced against the overall economics of the biocatalytic conversion.

Many thermophilic enzymes can be cloned and overexpressed in a soluble form using a mesophilic host (*Escherichia coli*) and can be easily purified from the cell extract by a straight forward heat treatment which precipitates most of the mesophilic proteins. The features responsible for the increased thermophilicity can be identified by studying the biochemical and structural features of a range of purified thermophilic proteins [10]. These include an increase in ionic interactions and often large ionic networks are observed within the protein and at the subunit interfaces. This is observed especially in hyperthermophilic proteins. The *α*-helices in the protein can be “capped” so that an acidic amino acid is placed to neutralise the charge of the protein at the amino end of the helix and a basic amino acid to neutralise the charge at the acidic end of the helix. Many thermophilic proteins have increased hydrophobicity within their interior and at subunit interfaces. This is especially true for thermophilic proteins from* Sulfolobus *species. They often have increased packing such as additional secondary structures and C-terminal extensions which can pack into the protein to fill unnecessary voids. Thermophilic proteins usually have shorter surface loops and often the internal loops can be stabilised by metal ions. An increased content of proline residues is seen in some thermophilic bacteria such as* Thermus* species which have a high G-C content in their DNA. Generally there is a reduction in amino acids that are unstable at high temperatures such as asparagines and cysteines except where they play an important catalytic role. Finally some species especially some of the aerobic archaea such as* Aeropyrum* species use the introduction of a covalent disulfide bond into the protein to offer the required stability to high temperature.

This paper will address some important industrially relevant biocatalytic reactions which can be carried out using archaeal enzymes.

## 2. Biocatalytic Industrial Applications

### 2.1. Production of Carbocyclic Nucleotides

**Scheme 1:**
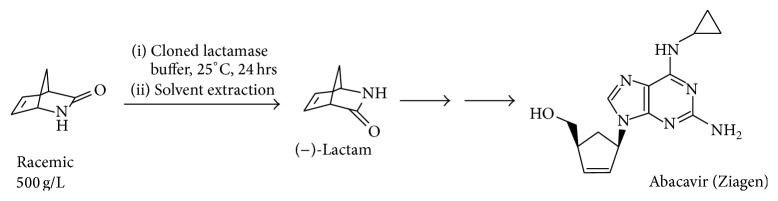
The lactamase catalytic process.

Carbocyclic nucleosides are valuable chemotherapeutic agents such as cardiac vasodilators and are used in the treatment of viral infection. New antiviral compounds are especially important for the treatment of HIV since they act as nucleotide inhibitors of the viral reverse transcriptase enzyme. The viral proteins can readily mutate in order to overcome the inhibition. New inhibitors therefore have to be continually developed. An enzyme from the archaeon* S. solfataricus* MT4 can use the bicyclic synthon (rac)-*γ*-lactam(2-azabicyclo[2.2.1]hept-5-en-3-one) as a substrate to obtain a single enantiomer of the *γ*-bicyclic lactam product which is an important building block for the anti-HIV compound, Abacavir (Scheme 1) [11]. This (+)-*γ*-lactamase was identified in the* Sulfolobus* strain by screening colonies from an expression library for their ability to produce the amino acid product when supplied with the racemic *γ*-lactam. Screening was carried out using genomic libraries using a filter paper overlay. The colonies on the plate that were active showed a brown colouration of the filter paper when the amino acid was produced which had been soaked in ninhydrin stain. Purified enantiomers of the (+) or (−)-*γ*-lactam were used as substrates to determine the stereospecificity of the enzyme. Another nonthermophilic bacterial (+)-*γ*-lactamase that can also carry out this reaction has been identified within the bacterium* Delftia acidovorans*. This enzyme is of a different class, structure, and mechanism from the archaeal enzyme but both can use the nonnatural *γ*-lactam as a substrate. This archaeal *γ*-lactamase has been cloned and overexpressed in* E. coli* and purified to homogeneity. The molecular mass of the monomer was estimated to be 55 kDa by SDS-PAGE which is consistent with the calculated molecular mass of 55.7 kDa. The native molecular mass was determined to be 110 kDa by gel filtration indicating that the enzyme exists as a dimer in solution. The purified enzyme has been crystallised with a view to determining its three-dimensional structure.

The thermostable archaeal *γ*-lactamase has a high sequence homology to the signature amidase family of enzymes. It shows similar inhibition patterns as the amidase enzymes towards benzonitrile, phenylmethylsulfonyl fluoride, and heavy metals such as mercury and it is activated by thiol reagents. The enzyme selectively cleaves the (+)-enantiomer from a *γ*-racemic mixture. It also exhibits general amidase activity by cleaving linear and branched aliphatic and aromatic amides [12, 13].

Alignment of the amino acid sequences of the *γ*-lactamase from* S. solfataricus* MT4 with 4 amidases from* Pseudomonas chlororaphis* B23,* Rhodococcus *sp. N-771,* R. erythropolis* N-774, and* Rhodococcus rhodochrous* J shows that it has a 41–44% sequence identity to these enzymes. The amidases belong to the signature amidase family as they all contain the consensus sequence GGSS(S/G)GS. The amino acid sequence of the *γ*-lactamase contains the highly conserved putative catalytic residues of aspartic acid and serine but not the highly conserved cysteine residue [14].

The purified (+)-*γ*-lactamase enzyme has been immobilised as a cross-linked, polymerised enzyme preparation and packed into microreactors [15]. The thermophilic (+)-*γ*-lactamase retained 100% of its initial activity at the assay temperature, 80°C, for 6 h and retained 52% activity after 10 h, indicating the advantage of the immobilisation. The high stability of the immobilised enzyme provided the advantage that it could be used to screen many compounds in a microreactor system without denaturation.

### 2.2. Transfer of Amine Groups

Many drugs are formed from chiral amines and there has been increasing interest from pharmaceutical companies to study the enzymes that can transfer amine groups from one compound to another in a stereospecific way. The transaminases catalyse the transfer of an amino group from an amino acid to a ketoacid [16]. They use the cofactor pyridoxal phosphate (PLP), the biologically active form of vitamin B6. The PLP normally covalently binds to an active site lysine amino acid by a Schiff base (internal aldimine). The mechanism of transaminases is made up of two half-reactions. In the first half-reaction the donor substrate gives its amino group to the cofactor, resulting in a keto acid and enzyme-bound pyridoxamine-phosphate (PMP). In the second half-reaction an amino group is transferred from PMP to an acceptor keto acid, producing an amino acid and restoring the PLP internal aldimine.

The archaeon* S. solfataricus* has been found to be an interesting source of a thermostable transaminase enzyme of group IV Pfam [17]. This pyridoxal phosphate containing enzyme is involved in the nonphosphorylated pathway for serine synthesis which is not found in bacteria and is found in animals and plants. The transaminase reaction that the enzyme carries out is the conversion of L-serine and pyruvate to 3-hydroxypyruvate and alanine. Activity is also shown towards methionine, asparagine, glutamine, phenylalanine, histidine, and tryptophan. The enzyme can be used in combination with transketolase for synthesis of chiral drug intermediates [18].

**Figure 1:**
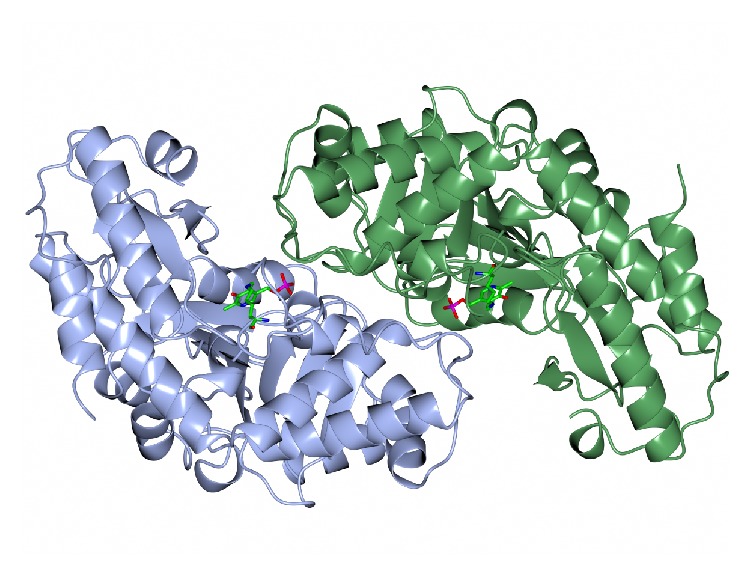
A ribbon diagram of the structure of the* Sulfolobus* transaminase dimer showing each monomer in a different colour as viewed along the molecular twofold axis. The cofactor pyridoxal phosphate (PLP) is shown bound to the active site lysine as a stick model in the two active sites (PDB code 3ZRP). The figure was constructed using CCP4mg [53].

The dimeric thermophilic archaeal transaminase enzyme structure has been solved in the holo form of the enzyme and in complex with an inhibitor gabaculine and in a substrate complex with phenylpyruvate, the keto product of phenylalanine [17].  Figure 1 shows a cartoon diagram of the dimeric* S. solfataricus* transaminase with the cofactors PLP in the two active sites.

The structural studies with this enzyme have given some insight into the conformational changes around the active site of the transaminase that occur during catalysis and have helped to understand the enzyme's substrate specificity. The most related enzyme to the* S. solfataricus* transaminase is the mesophilic yeast alanine:glyoxylate transaminase (AGAT) which shares 37% amino acid identity [19]. The yeast AGAT enzyme has been reported to have high substrate specificity for small amino acids such as alanine and glyoxylate, unlike the* Sulfolobus *transaminase which exhibits a broad amino acid substrate specificity. The substrate-binding pockets of AGATs are remarkably similar to that of* Sulfolobus* enzyme. The aromatic substrate phenylalanine was modelled into the* Sulfolobus* enzyme active site and was compared with the superimposed yeast AGAT enzyme structure. Most residues in the vicinity of the modelled substrate are conserved between the two proteins. The* Sulfolobus *enzyme has a significantly larger substrate-binding pocket as its loop region between strands 9 and 10 is two amino acids shorter. This gives more space for the bulkier phenylalanine substrate to bind compared with the AGAT enzyme, which is only active towards alanine and glycine. An isoleucine residue in AGAT occupies the space where larger substrates would sit in the active site of the* Sulfolobus* enzyme. The isoleucine is positioned 2.9 Å from the modelled substrate and hinders the binding of any amino acid larger than alanine. In the* S. solfataricus* transaminase enzyme there is a corresponding valine residue in the position of the isoleucine of AGAT which is positioned further away allowing the binding of larger amino acids. These subtle differences between the two enzymes are enough to change the enzymes substrate specificity which is vitally important to understand for its use in commercial applications.

**Figure 2:**
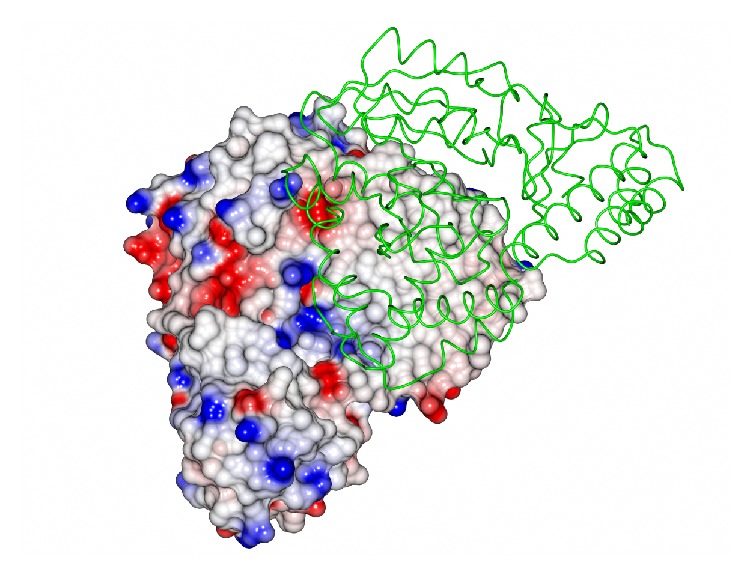
The structure of the* Sulfolobus* transaminase dimer showing one subunit in surface space filling mode where the white regions indicate hydrophobic regions and blue indicates basic regions and red acidic regions. The other subunit is represented as a *α*-C chain showing that the subunit interface is mainly hydrophobic (PDB code 3ZRP). The figure was constructed using CCP4mg [53].

The archaeal transaminase is relatively thermostable for 10 minutes at 70°C and at pH 6.5. Features of the archaeal enzyme that relate to its increased stability when compared with the related AGAT enzyme show that the yeast enzyme has 10 salt bridges compared to 21 salt bridges in the* Sulfolobus* transaminase which includes several 3-4 amino acid networks which offer increased stability. There is a C-terminal extension in the* Sulfolobus* enzyme and shorter surface loops which are all general features that are found in thermophilic enzymes. The* Sulfolobus* transaminase dimer interface is unusual being hydrophobic in nature with few ionic interactions which are generally associated with more thermophilic archaeal enzymes (Figure 2). This* Sulfolobus* serine transaminase is the first example of a thermophilic archaeal serine transaminase to be studied structurally and to show that its properties meet the requirements for the commercial application of the enzyme in biocatalysis.

### 2.3. Removal of Halogen Groups

**Figure 3:**
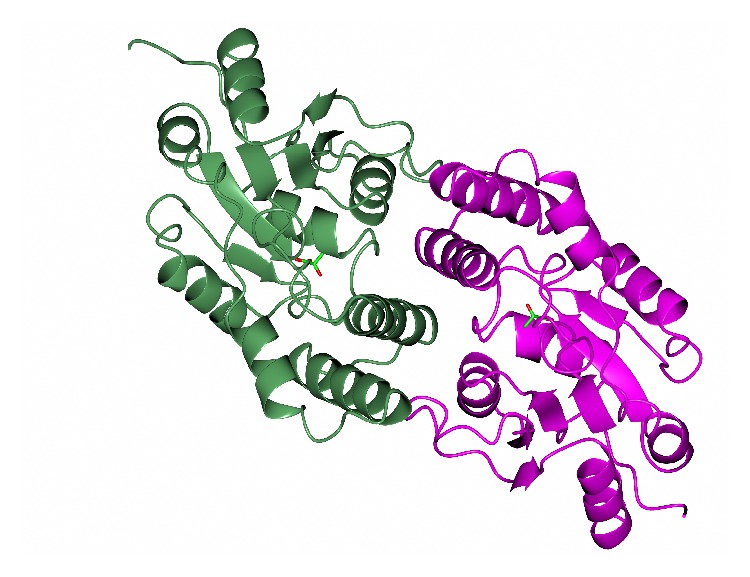
A ribbon diagram of the structure of the* Sulfolobus* dehalogenase dimer with each monomer shown in a different colour. The monomer has a core domain with a Rossmann-like fold of six-stranded parallel *β*-strands surrounded by five *α*-helices and three 3_10_ helices and a subdomain composed of *α*-helices. A substrate mimic is shown in the two active sites as a stick model, located between the two domains of each monomer (PDB code 2W11). The figure was constructed using CCP4mg [53].

Another archaeal enzyme of industrial interest is found in* Sulfolobus tokodaii.* This L-haloacid dehalogenase enzyme has been cloned and overexpressed in* E. coli*. It has been characterised biochemically and structurally [20, 21]. The enzyme monomer has two domains. The core domain has a Rossmann fold with a six-stranded parallel *β*-strand bundle surrounded by five *α*-helices and three 3_10_ helices. The subdomain is composed of *α*-helices. The active site is located between the two domains and the native enzyme forms a dimer as shown in Figure 3.

This enzyme has applications for chiral halo-carboxylic acid production and bioremediation. Chiral halo-carboxylic acids are important intermediates in the fine chemical/pharmaceutical industries. Removal of the halogen group can be carried out by a dehalogenase. The* Sulfolobus* enzyme has the potential to resolve racemic mixtures of bromocarboxylic acids. This L-bromoacid dehalogenase is able to catalyse the conversion of 2-halo-carboxylic acids to the corresponding hydroxyalkanoic acids. It has been shown to display activity towards longer chain substrates than the bacterial* Xanthomonas autotrophicus* dehalogenase [22] with activity seen towards 2-chlorobutyric acid. This is due to a more accessible active site. The enzyme has a maximum activity at 60°C and a half-life of over an hour at 70°C. It is stabilised by a salt bridge and hydrophobic interactions on the subunit interface, helix capping, a more compact subdomain than related enzymes, and shortening of surface loops. Another thermophilic enzyme of this family from hyperthermophilic archaea has addressed the problem of thermostability in a different way. The related* Pyrococcus* dehalogenase (29% sequence identity) whose structure is available from a structural genomics project is a monomeric enzyme stabilised by a disulfide bond [23].

### 2.4. Hydrolysis and Esterification

Esterases are a class of commonly used enzymes in industrial applications. This is partially due to their inherent stability in organic solvents and the ability to freely reverse the enzyme reaction from hydrolysis to synthesis by the elimination of water that is used in the hydrolysis mechanism. The carboxylesterases catalyse the hydrolysis of the ester bond of relatively small water soluble substrates. The carboxyl esterase NP originally identified from the fungus* Ophiostoma novo-ulmi* is used for the production of the nonsteroidal pain killing drug, naproxen [24]. The racemic naproxen methylester is hydrolysed to the* (S)*-acid which is separated from the* (R)*-methylester to yield* (S)*-naproxen with a 99% ee and a yield of 95%. It is important that new drugs coming to market are of one optical form preventing the problems associated with side effects of the inactive enantiomer.

A thermostable carboxylesterase from the archaeon* Sulfolobus shibatae* has been cloned, sequenced, and overexpressed in* E. coli *[25] (Toogood and Littlechild, unpublished data). The enzyme has a 71 to 77% sequence identity to an esterase from* S*.* tokodaii* and a carboxylesterase from* S. solfataricus* strain P1, respectively [26, 27]. This enzyme was identified as a serine esterase belonging to the mammalian hormone-sensitive lipase (HSL) family. It contains the conserved putative catalytic triad residues Ser, Asp, and His and is inhibited by the serine hydrolase inhibitors phenylmethylsulfonyl fluoride and benzamidine and partially inhibited by thiol reagents. The enzyme is thermostable, with no loss of activity detected after 24 h at 60°C. The enzyme was able to cleave a variety of* p*-nitrophenyl ester substrates, with the highest activity detected with* p*-nitrophenyl caproate. The carboxylesterase was also tested for its ability to cleave a variety of industrially relevant esters and diesters. It has a preference for substrates containing aromatic groups such as diethyl-2-benzyl malonate, benzyl acetoacetate, and Z-phenylalanine methyl ester. However, it was also able to enantioselectively cleave compounds such as 2-methyl-1,3-propanediol diacetate.

### 2.5. Resolution of Amino Acids and Amino Acid Analogues

Amino acids can be either of the “so-called” L configuration as found in all proteins or of the D configuration found in bacterial cell walls. The production of the specific L-amino acids and amino acid analogues is important for a variety of purposes. The industrial process to carry out this biocatalytic reaction makes use of an L-aminoacylase enzyme. A thermophilic archaeal version of this enzyme has been cloned and overexpressed from the archaeon* Thermococcus litoralis* [2]. The enzyme is a homotetramer of 43 kDa monomers and has an 82% sequence identity to an aminoacylase from* Pyrococcus furiosus* and 45% sequence identity to a carboxypeptidase from* S. solfataricus*. The enzyme is thermostable, with a half-life of 25 hours at 70°C. Cell-free extracts of the aminoacylase were found to have optimal activity at 85°C in Tris-HCl pH 8.0. Conventional aminoacylase inhibitors, such as mono-tert-butyl malonate, have only a slight effect on its activity. The* T. litoralis* L-aminoacylase has a broad substrate specificity preferring the amino acids: Phe ≫ Met > Cys > Ala ≃ Val > Tyr > Propargylglycine > Trp > Pro > Arg. A column bioreactor containing the recombinant* Thermococcus* L-aminoacylase immobilised onto Sepharose beads was constructed with the substrate, N-acetyl-DL-Trp, continuously flowing at 60°C for 10 days. No loss of activity was detected over five days, with 32% activity remaining after 40 days at 60°C [28]. The enzyme has also been immobilized into microreactors by covalent attachment to expoxy resins in channels of the reactor which allow the biocatalytic reaction to be carried out within this high throughput “flow” system [29]. This can be used for rapid screening of substrate specificity and eliminates problems with product inhibition often seen in industrial reactions which are carried out at high substrate concentrations. The* Thermococcus* L-aminoacylase enzyme is now being used in multiton commercial production of L-amino acids and their analogues by Chirotech/Dow Pharma and more recently by Chirotech/Dr. Reddy's for large scale biotransformations [30]. A racemase enzyme has been developed in order to convert the isomer not used by the enzyme to the form that is used which can enable a more efficient process with potentially 100% conversion of a racemic substrate [31].

The hyperthermophilic L-aminoacylase from* P. horikoshii* has also been cloned and overexpressed in* E. coli* [32]. There are differences in substrate specificity between the* Thermococcus* and the* Pyrococcus *enzymes. The substrate N-acetyl-L-phenylalanine is the most favourable substrate for the* Thermococcus* enzyme; however, this substrate is not used by the* Pyrococcus* L-aminoacylase.

### 2.6. Chiral Alcohol Production

**Figure 4:**
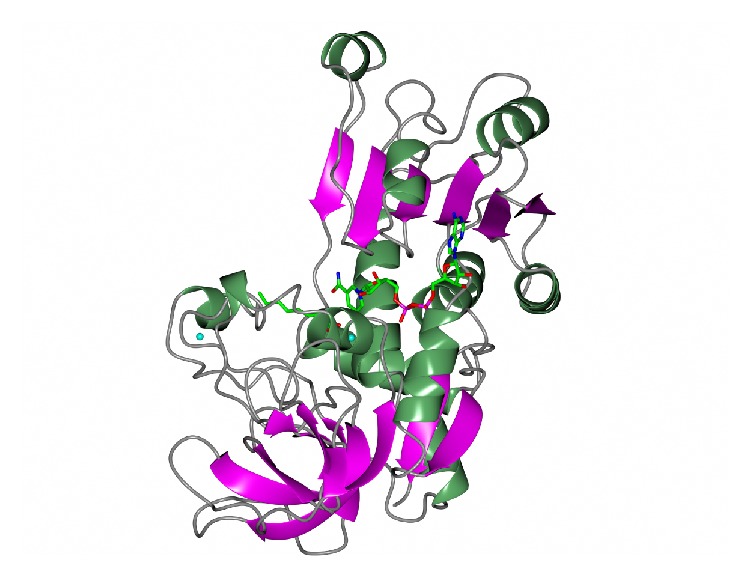
A ribbon diagram of the structure of the* Aeropyrum* alcohol dehydrogenase enzyme monomer showing the two different domains: the Rossmann domain which binds the NADH cofactor and the substrate-binding domain. The active site is located between the two domains where the cofactor and a substrate mimic are bound and shown as stick models. The two bound zinc ions are shown as blue spheres: the catalytic zinc in the active site and the structural zinc located in a distal loop. The active enzyme has a quaternary structure of a tetramer of four identical monomers (PDB code1H2B). The Figure was constructed using CCP4mg [53].

**Figure 5:**
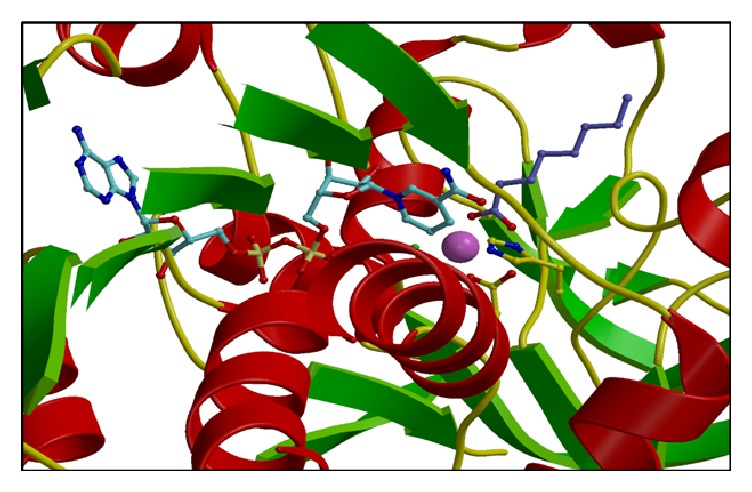
A ribbon diagram highlighting the details of the active site of the* Aeropyrum* ADH enzyme showing the catalytic zinc ion as a sphere together with the bound cofactor NADH and an enzyme inhibitor in stick mode, defining the substrate-binding pocket (PDB code 1H2B). The figure was constructed using CCP4mg [53].

**Figure 6:**
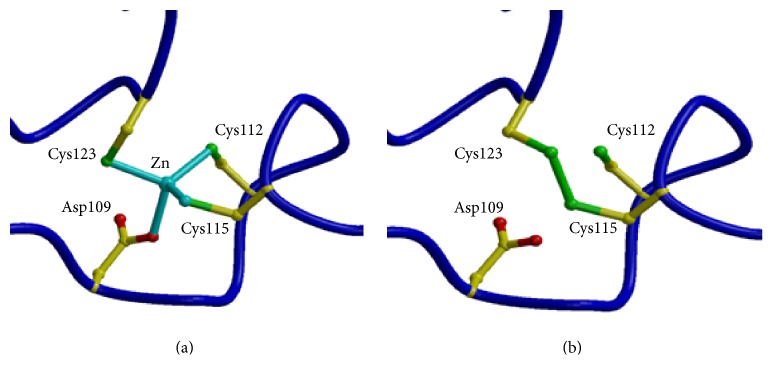
(a) The binding site of the structural zinc ion in the* Aeropyrum* ADH enzyme. (b) When zinc is not present a disulfide bond forms to stabilise the enzyme at this position in its structure (PDB code 1H2B). The figure was constructed with Bobscript [54].

A thermophilic aerobic archaeon* Aeropyrum pernix* is the source of a very stable alcohol dehydrogenase (ADH) enzyme that can be used for chiral alcohol production. This enzyme has been cloned and overexpressed in* E. coli* [33]. The* A. pernix* ADH enzyme is a tetrameric, zinc-containing, type I ADH with a monomer size of 39.5 kDa. It has sequence identity to a related horse liver ADH of 24% and the highest sequence identity to a known structure is 39% to a medium chain ADH from the hyperthermophilic archaeon* S. solfataricus* [34]. The* A. pernix* enzyme is highly specific for the cofactor NAD(H) and displays activity towards a broad range of alcohols, aldehydes, and ketones, while appearing to show a preference for cyclic substrates. The enzyme is very thermostable with a half-life of 2 hr at 90°C. The maximal activity is beyond 75°C; however, there is still 10% activity at 20°C. The enzyme is solvent stable with over 50% activity retained after incubation with 60% acetonitrile or dioxane. The crystal structure of the enzyme has been determined with an inhibitor bound into the active site [35]. The ADH monomer is formed from a catalytic and a cofactor binding domain, with the overall fold similar to previously solved ADH structures (Figure 4). The 1.62 Å resolution* A. pernix* ADH structure is that of the holo form, with the cofactor NADH bound into the cleft between the two domains. An inhibitor is bound in the active site which has been interpreted to be octanoic acid. This inhibitor is positioned with its carbonyl oxygen forming the fourth ligand of the catalytic zinc ion (Figure 5). The enzyme is stabilised by an ion-pair cluster at the subunit interfaces of the tetramer. There are two zincs bound to the enzyme, one at the active site and another at remote site which appears to stabilise the enzyme. When the zinc does not occupy this second site a disulfide bond is formed to hold the same two protein chains together (Figure 6). It has now been predicted that disulfide bonds do exist to stabilise many cytoplasmic proteins in this aerobic archaeon,* A. pernix *[36].

The enzyme is active against primary and secondary alcohols with optimum chain length of C4-C5. It is most active to large cyclic alcohols such as cycloheptanol and cyclooctanol. The enzyme reaction can be reversed to produce chiral alcohols by changing the pH. Initial experiments demonstrate that the* A. pernix* ADH shows some stereoselectivity in the reverse reaction producing the* (S)* phenylethanol [37] (Guy, 2002). The related* Sulfolobus* ADH has been reported to also be* (S)* selective showing enantiomeric excesses as high as 98% [38] (Raia et al., 2001).

Other thermophilic archaeal alcohol dehydrogenases have been characterised and are of the short chain or aldol keto reductase family such as the short-chain* Pyrococcus furiosus* ADH [39] and the aldol keto reductase* Thermococcus kodakarensis *ADH [40].

### 2.7. Cleavage of Lactone Rings

The specific cleavage of a lactone ring is an important activity of interest to the pharmaceutical companies. The lactonase enzymes identified to date fall into three structurally diverse groups: the enol lactonases, gluconolactonases, and the quorum sensing lactonases. Phosphotriesterase-like lactonases (PLLs) were identified in the archaeal species* S. solfataricus* and* S. acidocaldarius* [41–44]. These enzymes catalyse the hydrolytic cleavage of the intramolecular ester bond in lactones and acyl-homoserine lactones (AHLs) to give the corresponding hydroxyacylic acids. They also have a promiscuous but significantly lower phosphotriesterase activity towards organophosphate compounds. Recently an enzyme of this class has been identified, cloned, overexpressed, and characterised [45] from* Vulcanisaeta moutnovskia* a hyperthermoacidophilic crenarchaeon that was recently isolated from a solfataric field close to Moutnovsky volcano in Kamchatka (Russia) [46]. The VmutPLL converted lactones and acyl-homoserine lactones (AHLs) with comparable activities. A promiscuous, significantly lower activity was observed to organophosphates and only minor activity was observed with carboxylesters. The catalytic activity strictly depended on bivalent cations (Cd^2+^ > Ni^2+^ > Co^2+^ > Mn^2+^ > Zn^2+^). The VmutPLL showed a pH optimum around 8.0, a temperature optimum of 80°C, and a half-life of 26 min at 90°C. The enzyme has demonstrated high activity towards linear *γ*-lactones with hydrophobic side chains of variable lengths. These ranged from *γ*-butyrolactone (no side chain) and *γ*-valerolactone which has a methyl side chain and *γ*-dodecalactone which has a seven carbon side chain. It was shown that the enzyme has activity to whiskey lactone and *δ*-dodecalactone. No measurable activity was seen for mevalonolactone or *δ*-decalactone. For two of the substrates *γ*-valerolactone and *γ*-caprolactone, the optical isomeric forms of these were tested to determine stereoselectivity of the enzyme (Table 1). Results indicated that while activity is seen with both isomers the enzyme seems to favour the D form of these substrates.

The recent structure of the* V. moutnovskia* lactonase has been carried out in complex with a long chain fatty acid [47] which maps the substrate-binding pocket. This is lined by hydrophobic side chains, which would provide affinity for *γ*-lactones of any side chain length. The homologous lactonase from the* S. islandicus *is reported to favour *γ*-lactone substrates with long hydrophobic acyl chains of over four carbons in length [48]. The substrate-binding site of the* S. islandicus* lactonase revealed a number of polar residues at the entrance of the substrate pocket with hydrophobic residues lining the bottom of this pocket. This would disfavour the binding of the smaller *γ*-lactones in the correct catalytic position. The different distribution of hydrophobic and other residues in the active site pocket between the two related enzymes appears to be responsible for their different catalytic activities. The* V. moutnovskia* lactonase belongs to the amidohydrolase superfamily with a (*β*/*α*)_8_-barrel structural fold. In the* V. moutnovskia* enzyme these metals are two cobalt ions [48] that are essential for activity and are located in at the C-terminus of the *β* barrel. The coordinating residues of the metals are four histidines, one aspartate, and one lysine which are highly conserved in this enzyme family. In the proposed catalytic cycle the metal ions activate a bridging water molecule through proton abstraction. The resulting hydroxide ion then performs a nucleophilic attack on the C_1_ of the lactone ring resulting in hydrolysis [49].

The high thermal stability of this enzyme as well as its broad substrate specificity for different lactones AHLs and OPs makes it an interesting new enzyme for commercial biocatalysis. The enzyme is thought to have a natural role in quorum sensing which plays a role in some of the stages involved in biofilm formation [50, 51] and also in the expression of virulence factors that are of interest in medicinal and biotechnological applications [52]. The enzymatic degradation of AHLs catalysed by the PLLs provides potential for the interruption of quorum sensing signalling pathways and the control of microbial communities.

## 3. Conclusions

This review has summarised some of the important industrial applications using enzymes that have been identified from a variety of different thermophilic archaea. It is expected that the number of these enzymes used industrially will increase due to their inherent stability and novel specificities. The development of new rapid screening technologies and improved bioinformatic approaches in combination with new generation sequencing methodologies of archaeal genomes and metagenomes will provide new enzymes for industrial biotechnology. The enzymes can be cloned and overexpressed in easily grown hosts such as* E. coli* allowing access to sufficient quantities of the purified enzymes for detailed biochemical and structural characterisation. The scale-up of the enzyme production required for commercial applications can be carried out by using a fungal host system which allows export of the proteins into the growth medium for easy downstream processing. The cost of the enzyme biocatalyst is often the most expensive component of the industrial biotransformation and must be matched to the value of the end product. Higher value optically pure compounds which are important as drug intermediates for the pharmaceutical industries will allow a high enzyme price. Other enzymes that are required for the production of bulk chemicals, used as additives for domestic cleaning products, used in food production, or used to supplement biomass degradation processes, generally need to be marketed at a cheaper price and supplied in larger quantities. The stability of the biocatalyst is also an important issue since the enzyme ideally needs to be reused in several repeated biocatalytic cycles. Immobilisation of the enzyme can often increase its stability and allow it to be easily recovered for reuse. Thermostable enzymes are usually more robust under industrial conditions and can be used for repetitive biocatalytic conversions. The increased use of enzymes from the extremophilic archaea offers the opportunity to access biocatalysts that are naturally stable to a variety of different conditions of temperature, pH, salinity, and pressure making them better suited to different industrial processes.

The use of enzymes in “White Biotechnology” is expected to grow with biobased materials and chemicals produced from emerging technologies predicted to rise globally to over 7.4 million metric tons in 2018 (Lux Research Analysts). The initial process of enzyme discovery and optimisation is still a limiting factor in the adoption of new biobased industrial processes. There is therefore an increasing opportunity to commercialise newly discovered archaeal enzymes for sustainable manufacturing to contribute to the new circular economy.

## Figures and Tables

**Table 1:** The kinetic parameters of the activity of the VmutPLL enzyme with different lactone substrates. Taken from Kallnik et al., 2014 [45].

Substrate	Structure	Kcat [s^−1^]	Km [mM]	Kcat/Km [s^−1^ *∗*M^−1^]
*γ*-(*R*)-valerolactone	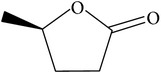	6.23 ± 0.42	4.56 ± 0.51	1327.1 ± 61.3
*γ*-(*S*)-valerolactone	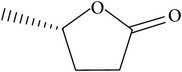	2.68 ± 0.00	1.95 ± 0.18	1379.95 ± 110.67
*γ*-(*R*)-caprolactone	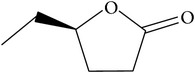	3.04 ± 0.01	0.55 ± 0.02	5563.72 ± 140.96
*γ*-(*S*)-caprolactone	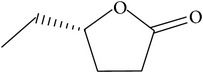	1.89 ± 0.11	0.75 ± 0.04	2531.22 ± 298.99
Whiskey lactone	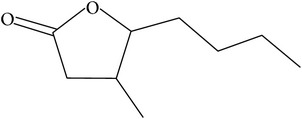	4.20 ± 0.06	0.93 ± 0.07	4538.63 ± 402.78
*γ*-Butyrolactone	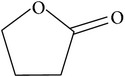	2.79 ± 0.07	11.57 ± 0.58	241.7 ± 11.18
pNP-acetate	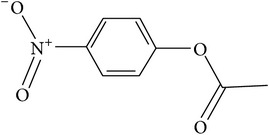	1.66 ± 0.34	8.19 ± 1.10	201.74 ± 20.48
Methyl-paraoxon	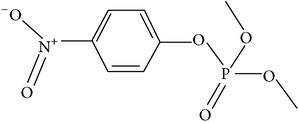	1.25 ± 0.40	2.79 ± 0.7	442.58 ± 50.14
